# Age, sex, and socioeconomic differences in multimorbidity measured in four ways: UK primary care cross-sectional analysis

**DOI:** 10.3399/BJGP.2022.0405

**Published:** 2023-01-10

**Authors:** Clare MacRae, Stewart W Mercer, David Henderson, Megan McMinn, Daniel R Morales, Emily Jefferson, Ronan A Lyons, Jane Lyons, Chris Dibben, David A McAllister, Bruce Guthrie

**Affiliations:** Advanced Care Research Centre, Usher Institute, College of Medicine and Veterinary Medicine, University of Edinburgh, Edinburgh, UK.; Advanced Care Research Centre, Usher Institute, College of Medicine and Veterinary Medicine, University of Edinburgh, Edinburgh, UK.; Centre for Population Health Sciences, Usher Institute, College of Medicine and Veterinary Medicine, University of Edinburgh, Edinburgh, UK.; Centre for Population Health Sciences, Usher Institute, College of Medicine and Veterinary Medicine, University of Edinburgh, Edinburgh, UK.; Division of Population Health and Genomics, University of Dundee, Dundee, UK; Department of Public Health, University of Southern Denmark, Denmark.; Health Informatics Centre, Population Health and Genomics, School of Medicine, University of Dundee, Dundee, UK.; Swansea University Medical School, Faculty of Medicine, Health and Life Science, Swansea University, Swansea, UK.; Swansea University Medical School, Faculty of Medicine, Health and Life Science, Swansea University, Swansea, UK.; School of Geosciences, College of Science and Engineering, University of Edinburgh, Edinburgh, UK.; Public Health, School of Health and Wellbeing, University of Glasgow, Glasgow, UK.; Advanced Care Research Centre, Usher Institute, College of Medicine and Veterinary Medicine, University of Edinburgh, Edinburgh, UK.

**Keywords:** epidemiology, multimorbidity, primary care, socioeconomic disparities

## Abstract

**Background:**

Multimorbidity poses major challenges to healthcare systems worldwide. Definitions with cut-offs in excess of ≥2 long-term conditions (LTCs) might better capture populations with complexity but are not standardised.

**Aim:**

To examine variation in prevalence using different definitions of multimorbidity.

**Design and setting:**

Cross-sectional study of 1 168 620 people in England.

**Method:**

Comparison of multimorbidity (MM) prevalence using four definitions: MM2+ (≥2 LTCs), MM3+ (≥3 LTCs), MM3+ from 3+ (≥3 LTCs from ≥3 International Classification of Diseases, 10th revision chapters), and mental–physical MM (≥2 LTCs where ≥1 mental health LTC and ≥1 physical health LTC are recorded). Logistic regression was used to examine patient characteristics associated with multimorbidity under all four definitions.

**Results:**

MM2+ was most common (40.4%) followed by MM3+ (27.5%), MM3+ from 3+ (22.6%), and mental–physical MM (18.9%). MM2+, MM3+, and MM3+ from 3+ were strongly associated with oldest age (adjusted odds ratio [aOR] 58.09, 95% confidence interval [CI] = 56.13 to 60.14; aOR 77.69, 95% CI = 75.33 to 80.12; and aOR 102.06, 95% CI = 98.61 to 105.65; respectively), but mental–physical MM was much less strongly associated (aOR 4.32, 95% CI = 4.21 to 4.43). People in the most deprived decile had equivalent rates of multimorbidity at a younger age than those in the least deprived decile. This was most marked in mental–physical MM at 40–45 years younger, followed by MM2+ at 15–20 years younger, and MM3+ and MM3+ from 3+ at 10–15 years younger. Females had higher prevalence of multimorbidity under all definitions, which was most marked for mental–physical MM.

**Conclusion:**

Estimated prevalence of multimorbidity depends on the definition used, and associations with age, sex, and socioeconomic position vary between definitions. Applicable multimorbidity research requires consistency of definitions across studies.

## INTRODUCTION

Multimorbidity is usually defined as the presence of ≥2 long-term conditions (LTCs).^1^ It is common in high-income countries, and is becoming more common in low- and middle-income countries.^1^ Multimorbidity poses major challenges to healthcare systems worldwide, and is associated with higher health service utilisation^2^ and mortality,^3^ but health services are usually designed to prioritise the management of single diseases.^4^ Definitions of multimorbidity are used inconsistently in research,^5^ and prevalence estimates vary widely across studies.^6^^,^^7^ This variation in prevalence is likely to relate not only to factors such as population demographics and study location, but also study methodology, including the definitions of multimorbidity used.^8^ Multimorbidity is known to be more prevalent in older people, females, and people with a lower socioeconomic position (SEP),^2^^,^^9^ but whether the strength of these associations depends on the definition used is uncertain.^10^

Some researchers have proposed that the conventional definition of multimorbidity as the presence of ≥2 LTCs does not capture those with the most complexity, disability, or functional impairment, and recommend using a higher cut-off, for example, ≥3 LTCs.^8^^,^^11^ Others suggest that complexity of management is better captured by defining multimorbidity in terms of multiple LTCs from multiple body systems (defined in terms of International Classification of Diseases, 10th revision [ICD-10] chapters).^12^ Coexistence of mental and physical health LTCs is also commonly suggested as a marker of complexity and need, and has been shown to be associated with higher levels of unplanned hospital admission^13^ and faster functional decline than physical-only multimorbidity.^14^ However, there has been little comparison of how the prevalence of multimorbidity in different population groups varies under different definitions.^15^

The aim of this study was to examine how prevalence of multimorbidity defined in four ways varied by age, sex, and SEP in a large primary care population in England.

**Table:** How this fits in

Multimorbidity poses major challenges to healthcare systems worldwide because of associated health service utilisation and mortality. Definitions with cut-offs in excess of ≥2 long-term conditions might better capture populations with complexity; however, these definitions are not standardised. Estimated prevalence of multimorbidity depends on the definition used, and associations with age, sex, and socioeconomic position vary between definitions. People in the most deprived decile had equivalent rates of multimorbidity at a younger age than those in the least deprived decile, and this difference was very large for those with mental–physical multimorbidity.

## METHOD

### Study design and data sources

This study used a cross-sectional design to examine variation in prevalence when measuring multimorbidity using four different definitions. The study population included people who were alive and registered with 149 Clinical Practice Research Datalink GOLD (CPRD)^16^ participating general practices in England on 30 November 2015, with 2 years of GP registration before the study index date.^17^ The study compared multimorbidity prevalence using four distinct definitions of multimorbidity, with the same 80 LTCs considered in the morbidity count for every analysis. Data were extracted from CPRD GOLD practices, including linked primary care and hospital data from electronic health records.

### Definition of outcomes and variables

For every individual, the authors of the current study defined the presence of 80 LTCs (10 of which were mental health conditions), categorised into ICD-10 chapters (Supplementary Table S1). The 80 conditions were chosen because they featured in phenotyping algorithms in the HDR-UK Phenotype Library,^18^ and/or were recommended by a recent Delphi consensus study^19^ and deemed to be relevant by the clinical authors (the first, second, and final authors). Codes used to identify individuals with each condition were mutually exclusive, therefore double counting of conditions was not possible. LTCs were defined using any code ever recorded in an individual’s record. This approach was applied to all 80 LTCs because the purpose of the study was to compare different cut-offs, therefore the authors chose to keep the method for defining the LTCs uniform. To do this, a set of existing code lists^18^^,^^20^ was used that combined Read codes (version 2) applied to GP electronic health record data, laboratory results recorded in the GP electronic health record, and also ICD-10 codes applied to hospital admission data to identify those at risk of poor outcomes^21^ (see Supplementary Table S2).

The study outcome in all analyses was the presence of multimorbidity, defined in four different ways. ‘Multimorbidity 2+’ was defined as the presence of ≥2 of the 80 LTCs and is the recommended definition.^1^ ‘Multimorbidity 3+’ was defined as ≥3 LTCs, ‘multimorbidity 3+ from 3+’ as ≥3 LTCs from ≥3 different ICD-10 chapters, and ‘mental– physical multimorbidity’ as the presence of ≥2 LTCs where ≥1 was a mental health LTC and ≥1 was a physical health LTC.

### Statistical analysis

The prevalence of multimorbidity using the four definitions was calculated, and associations with patient demographic characteristics — age at study index date, sex, and SEP (defined by Index of Multiple Deprivation [IMD] deciles)^22^ — were examined. Data for the characteristics of the study population were represented as counts and proportions with 95% confidence intervals (95% CIs). No data were missing for age, sex, or IMD decile. Logistic regression models were fitted to examine univariate (odds ratios [ORs]) and adjusted associations (adjusted odds ratios [aORs]), and 95% CIs of patient characteristics with the presence of multimorbidity using all four definitions. Multivariate models were adjusted for age, sex, and SEP. The large study size means that most comparisons were statistically significant, so clinical inference focused on the size of associations rather than *P*-values.

All analysis, modelling, and plotting was done in R (version 3.6.2) in the ISO27001 and Scottish Government-approved Health Informatics Centre Safe Haven environment.

## RESULTS

The study included 1 168 620 people with a median age of 44 years (IQR 23–60), of whom 587 687 (50.3%) were females, and 88 304 (7.6%) lived in the most deprived IMD decile areas (Table 1). There was substantial variation in the prevalence of multimorbidity using the four different definitions. In the whole study population (*n* = 1 168 620), multimorbidity 2+ had the highest prevalence (40.4%, *n* = 472 604), followed by multimorbidity 3+ (27.5%, *n* = 321 920), and multimorbidity 3+ from 3+ (22.6%, *n* = 264 035). Mental– physical multimorbidity had the lowest prevalence (18.9%, *n* = 220 774).

**Table 1.** Characteristics of the whole study population and cohorts defined by each definition of multimorbidity

**Characteristic**	**Whole population, n (% of whole population)**	**Population with multimorbidity 2+, n (row %)**	**Population with multimorbidity 3+, n (row %)**	**Population with multimorbidity 3+ from 3+, n (row %)**	**Population with mental–physical multimorbidity, n (row %)**
**Whole population**	1 168 620	472 604 (40.4)	321 920 (27.5)	264 035 (22.6)	220 774 (18.9)

**Age group, years**					
0–9	113 955 (9.8)	2741 (2.4)	617 (0.5)	527 (0.5)	448 (0.4)
10–19	137 517 (11.8)	9132 (6.6)	2437 (1.8)	1681 (1.2)	3432 (2.5)
20–29	122 237 (10.5)	24 919 (20.4)	9620 (7.9)	5081 (4.2)	13 614 (11.1)
30–39	143 243 (12.3)	39 875 (27.8)	18 809 (13.1)	10 560 (7.4)	23 154 (16.2)
40–49	176 061 (15.1)	66 748 (37.9)	36 590 (20.8)	24 782 (14.1)	38 405 (21.8)
50–59	173 435 (14.8)	89 267 (51.5)	57 029 (32.9)	45 043 (26.0)	46 731 (26.9)
60–69	141 041 (12.1)	98 234 (69.6)	72 820 (51.6)	62 694 (44.5)	42 985 (30.5)
70–79	97 843 (8.4)	82 596 (84.4)	69 649 (71.2)	62 824 (64.2)	30 101 (30.8)
≥80	63 288 (5.4)	59 092 (93.4)	54 349 (85.9)	50 843 (80.3)	21 904 (34.6)

**IMD decile**					
1 (least deprived)	167 558 (14.3)	62 032 (37.0)	40 593 (24.2)	33 343 (19.9)	25 820 (15.4)
2	129 704 (11.1)	51 504 (39.7)	34 680 (26.7)	28 625 (22.1)	22 291 (17.2)
3	128 234 (11.0)	51 794 (40.4)	35 047 (27.3)	29 016 (22.6)	22 917 (17.9)
4	109 986 (9.4)	45 681 (41.5)	31 143 (28.3)	25 822 (23.5)	20 588 (18.7)
5	127 816 (10.9)	53 601 (41.9)	36 775 (28.8)	30 377 (23.8)	24 051 (18.8)
6	104 158 (8.9)	44 279 (42.5)	30 545 (29.3)	25 325 (24.3)	20 753 (19.9)
7	108 782 (9.3)	44 097 (40.5)	30 291 (27.8)	24 812 (22.8)	21 220 (19.5)
8	103 501 (8.9)	43 102 (41.6)	29 717 (28.7)	24 295 (23.5)	21 813 (21.1)
9	100 577 (8.6)	40 019 (39.8)	27 471 (27.3)	22 107 (22.0)	20 784 (20.7)
10 (most deprived)	88 304 (7.6)	36 495 (41.3)	25 658 (29.1)	20 313 (23.0)	20 537 (23.3)

**Sex**					
Male	580 933 (49.7)	215 555 (37.1)	146 552 (25.2)	120 159 (20.7)	86 328 (14.9)
Female	587 687 (50.3)	257 049 (43.7)	175 368 (29.8)	143 876 (24.5)	134 446 (22.9)

*IMD = Index of Multiple Deprivation. LTCs = long-term conditions. Mental–physical multimorbidity = ≥2 LTCs where ≥1 mental health LTC and ≥1 physical health LTC are recorded. Multimorbidity 2+ = ≥2 LTCs. Multimorbidity 3+ = ≥3 LTCs. Multimorbidity 3+ from 3+ = ≥3 LTCs from ≥3 International Classification of Diseases, 10th revision chapters.*

**Figure 1.**
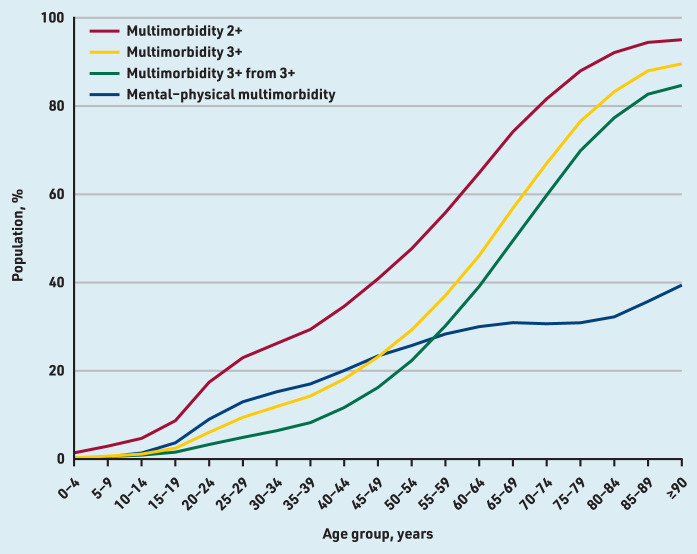
*Prevalence of multimorbidity by age using four different definitions. Multimorbidity 2+ = ≥2 long-term conditions (LTCs). Multimorbidity 3+ = ≥3 LTCs. Multimorbidity 3+ from 3+ = ≥3 LTCs from ≥3 International Classification of Diseases, 10th revision chapters. Mental–physical multimorbidity = ≥2 LTCs where ≥1 mental health LTC and ≥1 physical health LTC are recorded.*

Multimorbidity became more prevalent with increasing age when using all four definitions and showed an S-shaped relationship between prevalence and advancing age: a relatively slow increase in the youngest, rapid increases in adulthood, and flattening in later life (Figure 1). Multimorbidity 2+ had the highest prevalence in all age groups, and a faster rate of increase in early adulthood than multimorbidity 3+ and multimorbidity 3+ from 3+ (Table 1 and Figure 1).

Multimorbidity 2+, multimorbidity 3+, and multimorbidity 3+ from 3+ were strongly associated with oldest age (≥80 years versus 20–29 years); aOR 58.09 (95% CI = 56.13 to 60.14), aOR 77.69 (95% CI = 75.33 to 80.12), and aOR 102.06 (95% CI = 98.61 to 105.65), respectively. Mental– physical multimorbidity was much less strongly associated with oldest age (aOR 4.32, 95% CI = 4.21 to 4.43) (Table 2), but was present in more young and middle-aged adults than multimorbidity 3+ and multimorbidity 3+ from 3+ (Figure 1).

**Table 2.** Associations between patient characteristics and the presence of multimorbidity under four definitions

**Characteristic**	**aOR (95% CI) for multimorbidity 2+**	**aOR (95% CI) for multimorbidity 3+**	**aOR (95% CI) for multimorbidity 3+ from 3+**	**aOR (95% CI) for mental–physical multimorbidity**
**Age group, years^a^**				
0–9	0.10 (0.09 to 0.10)	0.06 (0.06 to 0.07)	0.11 (0.10 to 0.12)	0.03 (0.03 to 0.03)
10–19	0.28 (0.28 to 0.29)	0.22 (0.21 to 0.23)	0.29 (0.28 to 0.31)	0.21 (0.20 to 0.22)
20–29	Reference	Reference	Reference	Reference
30–39	1.53 (1.50 to 1.55)	1.80 (1.76 to 1.85)	1.87 (1.80 to 1.93)	1.55 (1.52 to 1.59)
40–49	2.50 (2.46 to 2.54)	3.25 (3.18 to 3.33)	3.98 (3.86 to 4.10)	2.34 (2.29 to 2.39)
50–59	4.39 (4.32 to 4.47)	6.16 (6.02 to 6.31)	8.62 (8.37 to 8.89)	3.14 (3.08 to 3.21)
60–69	9.60 (9.43 to 9.78)	13.62 (13.31 to 13.95)	20.31 (19.71 to 20.94)	3.75 (3.67 to 3.83)
70–79	22.75 (22.25 to 23.27)	31.82 (31.03 to 32.63)	45.06 (43.68 to 46.50)	3.77 (3.69 to 3.86)
* ≥*80	58.09 (56.13 to 60.14)	77.69 (75.33 to 80.12)	102.06 (98.61 to 105.65)	4.32 (4.21 to 4.43)

**IMD decile^b^**				
1 (least deprived)	Reference	Reference	Reference	Reference
2	1.10 (1.08 to 1.12)	1.12 (1.09 to 1.14)	1.11 (1.09 to 1.13)	1.12 (1.10 to 1.14)
3	1.16 (1.14 to 1.18)	1.18 (1.15 to 1.20)	1.17 (1.15 to 1.20)	1.19 (1.16 to 1.21)
4	1.20 (1.18 to 1.22)	1.22 (1.20 to 1.25)	1.21 (1.19 to 1.24)	1.24 (1.21 to 1.27)
5	1.25 (1.22 to 1.27)	1.28 (1.25 to 1.30)	1.25 (1.23 to 1.28)	1.26 (1.24 to 1.29)
6	1.33 (1.31 to 1.36)	1.38 (1.36 to 1.41)	1.37 (1.34 to 1.40)	1.38 (1.35 to 1.41)
7	1.37 (1.34 to 1.39)	1.46 (1.43 to 1.49)	1.43 (1.40 to 1.47)	1.43 (1.40 to 1.46)
8	1.52 (1.50 to 1.55)	1.63 (1.59 to 1.66)	1.59 (1.56 to 1.63)	1.63 (1.59 to 1.66)
9	1.63 (1.60 to 1.66)	1.80 (1.76 to 1.84)	1.74 (1.70 to 1.78)	1.72 (1.69 to 1.76)
10 (most deprived)	1.93 (1.89 to 1.97)	2.23 (2.18 to 2.28)	2.09 (2.04 to 2.14)	2.14 (2.10 to 2.19)

**Sex^c^**				
Male	Reference	Reference	Reference	Reference
Female	1.31 (1.30 to 1.32)	1.19 (1.18 to 1.20)	1.15 (1.14 to 1.16)	1.70 (1.68 to 1.71)

a *Adjusted for socioeconomic position and sex.*

b *Adjusted for age and sex.*

c *Adjusted for age and socioeconomic position. aOR = adjusted odds ratio. IMD = Index of Multiple Deprivation. LTCs = long-term conditions. Mental–physical multimorbidity = ≥2 LTCs where ≥1 mental health LTC and ≥1 physical health LTC are recorded. Multimorbidity 2+ = ≥2 LTCs. Multimorbidity 3+ = ≥3 LTCs. Multimorbidity 3+ from 3+ = ≥3 LTCs from ≥3 International Classification of Diseases, 10th revision chapters.*

Using all four multimorbidity definitions, prevalence (Table 1) and aORs (Table 2 and Supplementary Tables S3–S6) of multimorbidity were higher in the most versus least deprived IMD decile: multimorbidity 2+ aOR 1.93 (95% CI = 1.89 to 1.97), multimorbidity 3+ aOR 2.23 (95% CI = 2.18 to 2.28), multimorbidity 3+ from 3+ aOR 2.09 (95% CI = 2.04 to 2.14), and physical–mental multimorbidity aOR 2.14 (95% CI = 2.10 to 2.19).

**Figure 2.**
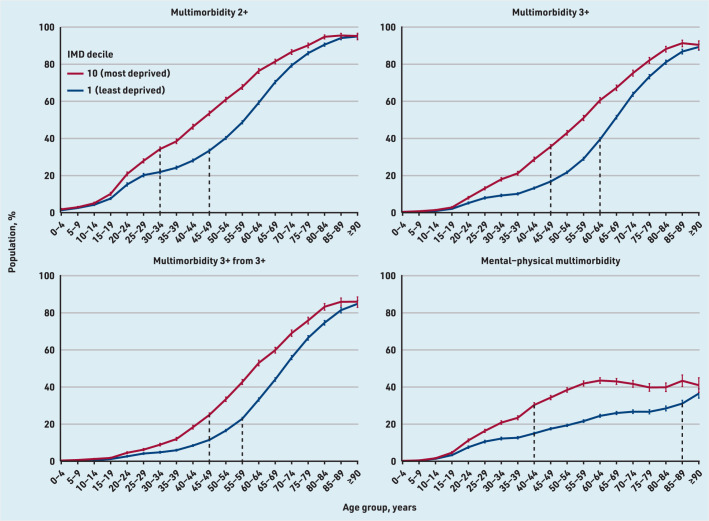
*Prevalence of each definition of multimorbidity in the most and least deprived IMD decile, by age. Graphical representation of the estimated multimorbidity prevalence for each of the four definitions, comparing the most and least deprived IMD decile. 95% confidence intervals are represented by coloured vertical lines. Dashed vertical black lines represent the point at which the horizontal gap (difference in multimorbidity prevalence) between most and least deprived IMD deciles is largest (that is, where there is greatest inequality in the age at which people have multimorbidity). IMD = Index of Multiple Deprivation. Multimorbidity 2+ = ≥2 long-term conditions (LTCs). Multimorbidity 3+ = ≥3 LTCs. Multimorbidity 3+ from 3+ = ≥3 LTCs from ≥3 International Classification of Diseases, 10th revision chapters. Mental–physical multimorbidity = ≥2 LTCs where ≥1 mental health LTC and ≥1 physical health LTC are recorded.*

Figure 2 shows multimorbidity prevalence in the most and least deprived IMD deciles by age: multimorbidity was more prevalent in the most deprived decile at all ages, with a widening of the gap starting at adolescence, widest in middle age, and converging in the oldest age group. There was a stepwise increase in multimorbidity prevalence with each IMD decile from least to most deprived for all definitions (Supplementary Table S7 and Supplementary Figures S1–S4).

Females had higher prevalence (Table 1) and aORs (Table 2) of multimorbidity versus males using all four definitions, although absolute differences were modest for multimorbidity 2+ (aOR 1.31, 95% CI = 1.30 to 1.32), and very small for multimorbidity 3+ (aOR 1.19, 95% CI = 1.18 to 1.20) and multimorbidity 3+ from 3+ (aOR 1.15, 1.14 to 1.16). The largest differences were observed for mental–physical multimorbidity (prevalence of 22.9% [ *n* = 134 446/220 774] in females and 14.9% in males [ *n* = 86 328/220 774]; aOR 1.70, 95% CI = 1.68 to 1.71 in females versus males).

Figure 2 shows the widest horizontal gap between multimorbidity prevalence by age between the most and least deprived IMD deciles for each definition of multimorbidity. In multimorbidity 2+, the widest age gap (horizontal distance between the most and least deprived IMD deciles) was 15–20 years, that is, people in the most deprived IMD decile had similar prevalence of multimorbidity 15–20 years younger than those in the least deprived IMD decile.

For multimorbidity 3+ and multimorbidity 3+ from 3+ the widest age gap in multimorbidity prevalence was 10–15 years. However, a much larger difference of 40–45 years was seen in mental–physical multimorbidity; 34.3% of those aged 45–49 years in the most deprived IMD decile had mental–physical multimorbidity versus 31.1% of those aged 85–89 years in the least deprived (Figure 2 and Supplementary Table S3).

## DISCUSSION

### Summary

This study found substantial variation in the prevalence of multimorbidity using four different published definitions of multimorbidity. Multimorbidity 2+ had the highest prevalence, followed by multimorbidity 3+ and multimorbidity 3+ from 3+, and in all these definitions prevalence was considerably higher with increasing age. The prevalence was lowest using the mental–physical multimorbidity definition, with a flatter age distribution and higher prevalence in younger to early middle-aged adults than using the multimorbidity 3+ and multimorbidity 3+ from 3+ definitions. Multimorbidity prevalence was higher in people living in more deprived areas, and for all definitions inequalities (the difference in prevalence between the most and least deprived groups for each definition) were largest in middle age. At the point of greatest difference, people in the most deprived IMD decile would have the same prevalence of multimorbidity 40–45 years younger using the mental–physical multimorbidity definition, 15–20 years younger using the multimorbidity 2+ definition, and 10–15 years younger using the multimorbidity 3+ and multimorbidity 3+ from 3+ definitions. Prevalence of multimorbidity was higher in females than males using all four definitions, although adjusted associations were weak for all but mental–physical multimorbidity.

### Strengths and limitations

Strengths of this study include systematic analysis of multimorbidity prevalence rates using a large primary care population dataset. Multimorbidity prevalence calculations using each definition were based on counting 80 LTCs (compared with a median of 17 LTCs reported in the wider literature^10^), including 10 mental health conditions, and almost all the conditions recommended by a recent international Delphi consensus study.^19^ The study, however, has a number of limitations. The dataset marginally underrepresents people in the most deprived IMD decile (7.6% versus 10% of the population of England). A mitigating factor is the large population size, which provides improved accuracy in the estimation of variance between associations with IMD deciles and stratification by SEP. All conditions were counted as equivalent, with no weighting based on severity, impact on quality of life, or clinical outcomes. However, unweighted counts are appropriate when the purpose is to measure prevalence,^19^ and future research could usefully explore associations of different multimorbidity measures with patient outcomes. The study uses routinely collected data, and given that these data are not collected for research purposes, errors and biases can be introduced at the collection and cleaning stages because of issues such as underreporting, data-linkage problems, and misclassification bias.^23^ However, because these data were collected under real-world conditions they maximise representativeness and generalisability of the population studied, and allow examination of a large population size.^23^

### Comparison with existing literature

Some existing literature examines different definitions of multimorbidity. Storeng *et al*^11^ used patient self-report of 38 conditions in people aged 60–69 years in Norway to examine multimorbidity defined as the presence of ≥3 LTCs from ≥3 ICD-10 chapters, termed ‘complex multimorbidity’ (the same definition as multimorbidity 3+ from 3+ in the current study). Multimorbidity 3+ from 3+ was present in 47.8% of those aged 60–69 years, which is close to the 44.5% estimate in this study, although they found larger differences in prevalence between females and males. Multimorbidity 3+ from 3+ was strongly associated with the need for assistance with activities of daily living and moderately associated with mortality.^11^

Kato *et al*^12^ performed a population-based study in Japan examining multimorbidity 2+ and multimorbidity 3+ from 3+ (also termed ‘complex multimorbidity’) in 38 889 people who were both functionally independent and not receiving any nursing care when they completed a self-report questionnaire. Multimorbidity 2+ prevalence for people aged ≥65 years was 52.0%, which is lower than the estimates in this study (69.6% in those aged 60–69 years rising to 93.4% in those aged ≥80 years). Similarly, prevalence of multimorbidity 3+ from 3+ was lower in people aged ≥65 years; 19.5% in people aged ≥65 years compared with prevalence of 44.5% in those aged 60–69 years rising to 80.3% in those aged ≥80 years in the present study. These differences are likely to reflect the selection of healthier people in the Japanese study; however, significant associations were also observed between both multimorbidity 2+ and multimorbidity 3+ from 3+ with mortality.

Socioeconomic deprivation was significantly associated with multimorbidity in a Scottish study by Barnett *et al*,^9^ who reported prevalence rates of 11.0% in the most deprived area versus 5.9% in the least deprived area. Similarly, Payne *et al*^13^ performed a retrospective cohort study in Scotland and found that mental health morbidity was more prevalent in areas of deprivation and independently associated with increased rates of unplanned hospital admission. Hauswaldt *et al*^24^ examined prevalence of multimorbidity 2+ and multimorbidity 3+ from 3+ in German general practices. They found that females were more likely to be multimorbid than males and the sex ratio remained stable across both definitions; however, they did not examine mental–physical multimorbidity, which in the current study was more strongly associated with being a female than the other definitions.

### Implications for research and practice

There are several areas that require further research. Large studies examining the relationship between multimorbidity types with important outcomes (such as functional status and quality of life, unscheduled hospital admission, and death) are needed because different definitions may be appropriate to facilitate targeting of particular groups of patients for intervention. This might be particularly important for mental and physical health combinations where mental health inequalities are a large driver for the difference in multimorbidity prevalence rates between most and least deprived categories using that definition. Further exploration of problems experienced by people with each definition of multimorbidity is needed, including issues relating to access to and continuity of care, so that services and interventions can be better designed to meet the needs of people with all definitions of multimorbidity.^25^

This work builds understanding of disparities in the prevalence of multimorbidity based on age, sex, and, most strikingly, SEP, using different definitions. A key recommendation from a recent systematic review of systematic reviews of the definition and measurement of multimorbidity is that researchers need to be explicit about the definition used and rationale for this choice, so that comparisons can be made across studies from different settings.^26^ Using a cut-off of ≥2 LTCs will allow researchers to be consistent with the majority of existing research;^10^^,^^26^ however, the current study shows that a markedly different population group is identified when using the mental–physical multimorbidity definition compared with this most common definition. This is important because clinical judgement is required to adapt care accordingly for people with multimorbidity where the patient experience can involve difficulties managing competing treatment demands, especially seen in people with coexisting mental and physical health LTCs (for example, the additional difficulties experienced by people with schizophrenia and cardiovascular disease and/or diabetes where the condition itself can affect the ability to engage in lifestyle changes, and treatment with antipsychotics additionally predispose to cardiovascular risk).^27^ Therefore, alternative definitions of multimorbidity, such as mental–physical, might be used to redistribute allocation of resource to general practices in areas of higher deprivation, and within these practices, towards a markedly younger population than in areas of lower deprivation where the age distribution of mental–physical multimorbidity is very different. Therefore, GPs can promote bespoke clinical judgements and reach shared care goals about a person’s needs, preferences, and health priorities^28^ for this group who are known to have worse clinical outcomes than those with physical-only multimorbidity.^13^^,^^14^

People living in the most deprived areas experience a greater burden of multimorbidity across all definitions, with a consistent dose–response effect (Supplementary Figures S1–S4), and this is most marked for mental–physical multimorbidity. Therefore, it is essential that policy and funding decisions support recommendations to tackle the inverse care law^29^ and coordinate services to target higher-need populations, providing care delivered by multidisciplinary teams in these communities, with integration of health and social care services, and a particular focus on delivering combined care for mental and physical health conditions.^30^ Additionally, continued work is needed to support GPs with appropriate clinical decision-making tools and models of care to support them in managing individuals with multiple conditions.^28^

In conclusion, this study finds that different definitions of multimorbidity have varying associations with age, sex, and SEP. Understanding which people in society have higher rates of different definitions of multimorbidity can help GPs and policymakers to plan provision of care. Establishment of international consensus over which multimorbidity definitions should be used, in both research and clinical contexts, will improve translation of research findings across studies and provide clinical benchmarking to aid identification of individuals who are more likely to require additional support.

## Data

CPRD are the data controller, and the data used for analysis cannot be shared under the licence granted to the authors.
